# A systematic literature review of automatic Alzheimer’s disease detection from speech and language

**DOI:** 10.1093/jamia/ocaa174

**Published:** 2020-09-14

**Authors:** Ulla Petti, Simon Baker, Anna Korhonen

**Affiliations:** Department of Theoretical and Applied Linguistics, University of Cambridge, Language Technology Lab, Cambridge, UK

**Keywords:** Alzheimer’s disease, dementia, natural language processing, speech, language

## Abstract

**Objective:**

In recent years numerous studies have achieved promising results in Alzheimer’s Disease (AD) detection using automatic language processing. We systematically review these articles to understand the effectiveness of this approach, identify any issues and report the main findings that can guide further research.

**Materials and Methods:**

We searched PubMed, Ovid, and Web of Science for articles published in English between 2013 and 2019. We performed a systematic literature review to answer 5 key questions: (1) What were the characteristics of participant groups? (2) What language data were collected? (3) What features of speech and language were the most informative? (4) What methods were used to classify between groups? (5) What classification performance was achieved?

**Results and Discussion:**

We identified 33 eligible studies and 5 main findings: participants’ demographic variables (especially age ) were often unbalanced between AD and control group; spontaneous speech data were collected most often; informative language features were related to word retrieval and semantic, syntactic, and acoustic impairment; neural nets, support vector machines, and decision trees performed well in AD detection, and support vector machines and decision trees performed well in decline detection; and average classification accuracy was 89% in AD and 82% in mild cognitive impairment detection versus healthy control groups.

**Conclusion:**

The systematic literature review supported the argument that language and speech could successfully be used to detect dementia automatically. Future studies should aim for larger and more balanced datasets, combine data collection methods and the type of information analyzed, focus on the early stages of the disease, and report performance using standardized metrics.

## INTRODUCTION

Dementia affects around 50 million people worldwide, and, due to population aging, the number of dementia sufferers is expected to triple in the next 30 years.^1^ Alzheimer’s disease (AD) is the most common neurodegenerative disease contributing to 60%–70% of dementia cases^1^ and affecting 1 in 14 people over the age of 65 and 1 in 6 people over the age of 80.^2^

Detecting AD is often challenging as clear manifestations often don’t appear until several years after onset. Diagnosing dementia can be costly and time-consuming, as it requires access to a qualified clinician. Both factors contribute to 55% of dementia cases remaining undiagnosed in the US.^3^

In recent years, numerous studies have suggested that language dysfunction is 1 of the earliest signs of cognitive decline,^4–6^ enabling the features of language and speech to act as biomarkers in early dementia detection.^7–9^

Memory impairment typical in AD contributes to many of these dysfunctions. For example, word retrieval difficulties may be the earliest signs of AD,^10^ manifesting in changes in several language aspects, such as verbal naming,^11^ speech content density and quantity,^12^ accurate meaning communication,^4^ pausation, and speech tempo.^13^^,^^14^ Word retrieval is often tested using picture description tasks^15^ where the participants are instructed to describe what they see in a picture. In addition to word retrieval processes, these tasks allow assessing lexical and syntactic complexity, the decline of which has also been reported in dementia.^5^^,^^16^

Memory deficit also contributes to the tendency to repeat words and concepts which can result in communication errors, lower coherence, and information density.^17^ Repetitions can manifest in spontaneous speech or fluency tests. Typical fluency tests are semantic verbal fluency task (SVF) and phonemic verbal fluency task (PVF) where the participants are asked to name as many words in 1 minute as they can that are either from the same semantic category (SVF) or begin with the same letter (PVF). SVF tasks also allow assessing how semantic information is accessed, which is 1 of the most severely affected language areas in dementia.^6^^,^^18–22^

While until recently language data were analyzed manually, the development of technology has enabled automating the analysis. Automation promotes the inclusion of more data and more detailed analysis revealing patterns that may go unrecognized in manual analysis. Promising results have been achieved in AD detection using natural language processing (NLP), signal processing (SP), and machine learning (ML). NLP is concerned with understanding, learning, and producing human language using computational tools.^23^ SP explores signals and the information they convey and is concerned with how they can be transformed, manipulated, and represented.^24^ ML focuses on the questions concerned with constructing computer programs that can improve automatically based on experience.^25^

Automating language processing could provide a noninvasive and fast approach to detecting clinical conditions and making screening for dementia accessible and affordable. A successful tool would allow people with limited access to healthcare to screen at home for early signs of dementia using, for example, a mobile application. Automating the analysis of language tests could also benefit clinicians during in-hospital screenings. While these technologies would be useful, they are still in the development stage and are not yet publicly available.

This systematic literature review aims to provide a comprehensive overview of the state-of-the-art of automatic dementia detection from language and speech and identify the best practices and the main challenges to guide further research on the topic.

## OBJECTIVES

We aim to systematically analyze 5 key questions: (1) What were the characteristics of the participant groups involved in the studies? (2) What type of language data were collected and how? (3) Which were the most informative language and speech features? (4) What classification methods were used? (5) What classification performance was achieved? These questions are helpful to clinicians and researchers because they help to identify best practices, summarize the state-of-the-art in automatic language processing for dementia detection, and guide further research.

## MATERIALS AND METHODS

The review protocol followed is the Preferred Reporting Items for Systematic Reviews and Meta-Analyses (PRISMA) checklist.^26^

### Search process

We searched the 3 largest databases: PubMed, Web of Science, and Ovid, using the keywords 1) automatic Alzheimer’s disease detection, 2) Alzheimer natural language processing, 3) Alzheimer speech processing. All articles were published between 2013 and 2019 to allow capturing the most recent literature and focusing on the time period where NLP, SP, and ML have been increasingly used in disease detection from speech and language. The last search date was August 8, 2019.

### Selection process

We established the following inclusion criteria in all studies: 1) AD or MCI was the condition of at least 1 of the participants, 2) participants’ language or speech was considered, 3) there was either an NLP, SP, or ML element; 4) the focus was on language or speech production, and not comprehension; 5) experimental data were included; 6) full articles were available in English. Initial study selection was performed by 1 reviewer (UP). To minimize the bias in selecting studies, a sample of 274 articles consisting of a random sample of 10% of the articles excluded by the first reviewer (n = 241), and all the articles included by the first reviewer (n = 33) were independently reviewed by a second reviewer (SB). The initial overall agreement between the 2 reviewers was 97%, with 100% agreement on the 33 included articles. Remaining disagreement was resolved in a discussion with the third author (AK).

### Data extraction and synthesis

The following data relevant to the 5 research questions were extracted from all included articles: participant information, the type of language data and the language tests used, the most informative language and speech features, classification methods, and classification performance.

## RESULTS

### Study selection

**Figure 1.**
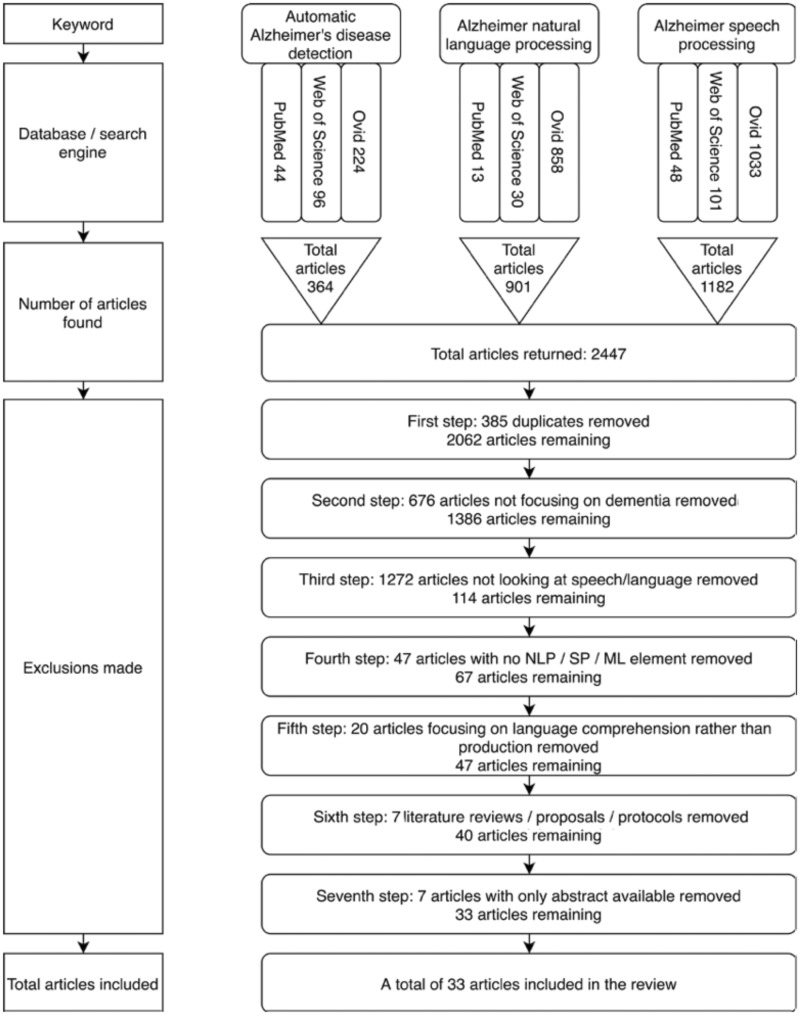
Flow diagram of study selection.

The number of articles retrieved from the initial search was 2447. The flow diagram displayed in Figure 1 details the selection process that resulted in 33 included articles.

### Study characteristics

Out of 33 studies, 18 focused on AD, 9 on both AD and MCI, and 6 solely on MCI. Twenty- 8 studies focused on spontaneous speech (SS), and 7 on both verbal fluency tasks (VF) and other tasks (OT). On average, 92 participants were included in the studies, with the number of participants ranging from 3 to 484. One study only reported the number of recordings^7^ and all but 2 studies^27^^,^^28^ had a healthy control group. The average size of the control group was 43, ranging from 2 to 242,^30^ and the average size of the AD group was 45, the MCI group was 30, and the dementia group was 27. A large majority of the studies were conducted in European languages: 10 studies in English, 4 in French and Hungarian, 3 in Greek and Turkish, and 1 in Spanish and Italian. One study was carried out with Taiwanese speakers, 5 studies used a dataset consisting of several languages, and 4 studies did not specify the language used. The number of studies grew year by year; 3 studies were published in 2013, 5 studies in 2014, 2015 and 2016, 6 studies in 2017, and 9 studies in 2018. This shows that research in the area is growing.

The information extracted from the 33 studies is summarized in Table 1.

**Table 1.** Information extracted from the studies

No	Study	Impaired group size (s)	Control group size	AD/MCI	Data collection method	Most informative language and speech features	Number of samples used to train the model	Classification algorithm	Classification performance
1	Ammer and Ayed 2018^30^	AD n = 242	n = 242	AD	SS	Repetition, word errors, MLU morphemes, POS	–	SVM, NN, DT	Precision = 79%
2	Beltrami et al 2018^31^	aMCI n = 16, mdMCI n = 16, early dementia n = 16	n = 48	MCI	SS	Acoustic, lexical, syntactic	–	–	–
3	Boye et al 2014^32^	AD n = 5	n = 5	AD	SS	Lexical and semantic deficit, reduced conversation	–	–	–
4	Chien et al 2018^33^	1) AD n = 15 2) AD n = 30	1) n = 15 2) n = 30	AD	SS	Speech length, non-silence tokens	150 samples from 60 participants	RNN	AUC = 0.956
5	Clark et al 2014^34^	MCI n = 23, AD n = 10	n = 25	AD, MCI	SVF, PVF	Semantic similarity of words	–	–	–
6	Clark et al 2016^35^	MCI-con n = 24, MCI-non n = 83	n = 51	MCI	SVF, PVF	Coherence, lexical frequency, graph theoretical measures	158	Random forest, SVM, NB, MLP	Acc = 81–84%
7	Fang et al 2017^29^	MCI-con n = 1	n = 2	MCI	SS	Unique and specific words, grammatical complexity	–	–	–
8	Fraser et al 2016^8^	AD n = 167	n = 97	AD	SS	Semantic, acoustic, syntactic, information	473 samples from, 264 participants	Logistic regression	Acc = 81%
9	Garrard et al 2017^28^	Probable AD n = 5	n = 0	AD	SS	–	–	–	–
10	Gosztolya et al 2016^36^	MCI = 48	n = 36	MCI	SS	Filled pauses	84	SVM	Acc = 88.1%
11	Gosztolya et al 2019^37^	MCI n = 25, early AD n = 25	n = 25	AD, MCI	SS	Semantic, morphological, acoustic attributes	75	SVM	Acc = 86%
12	Guinn et al 2014^38^	AD n = 28	n = 28	AD	SS	Ratio, POS, lexical, pauses, fillers	56	NB, DT	precision = 80%
13	Hernandez-Dominguez et al 2018^39^	AD n = 169 MCI n = 19	n = 74	AD, MCI	SS	Information coverage, auxiliary verbs, hapax legomena	236 training, 26 testing	SVM, Random Forest	Acc = 87–94%
14	Khodabakhsh et al. 2014a^40^	AD n = 27	n = 27	AD	SS	Log voicing ratio, average absolute delta formant and pitch	54	SVM, DT	Acc = 88–94%
15	Khodabakhsh et al. 2014b^41^	AD n = 20	n = 20	AD	SS	Fillers, unintelligibility, no of words, confusion, pause & no answer rate	40	SVM, DT	Acc = 90%
16	Khodabakhsh et al 2015^42^	AD n=28	n=51	AD	SS	Ratio, POS, speech rate features	79	SVM, NN NB, CTree	Acc = 84%
17	Konig et al 2015^43^	MCI n=23, AD n=26	n=15	AD, MCI	SS, SVF, OT	Speech continuity, ratio	64	SVM	EER = 13–21%
18	Konig et al 2018^27^	AD n=27, mixed dementia n=38, MCI n=44, SCI n=56	n=0	AD, MCI	SS, SVF, PVF, OT	Location of first word, words’ distribution in time	165	SVM	Acc = 86%
19	Lopez-de-Ipina et al 2013a^44^	AD n = 20	n = 20	AD	SS	Impoverished vocabulary, limited replies	40	MLP	Acc = 75–94.6%
20	Lopez-de-Ipina et al 2013b^45^	Early AD n = 1 Intermediate AD n = 2 Advanced AD n = 2	n = 5	AD	SS	Fluency, acoustic	10	SVM, MLP, kNN, DT, NB	Acc = 93.79%
21	Lopez-de-Ipina et al 2015^46^	AD n = 20	n = 20	AD	SS	Duration, time, frequency	40	MLP, KNN	Acc = 95%
22	Lopez-de-Ipina et al 2018^47^	1) AD n = 6, 2) AD n = 20, 3) MCI n = 38	1) n = 12, 2) n = 20 3) n = 62	AD, MCI	SS, SVF	Voicing, pauses, F0, harmonicity	18, 40, 100	MLP, CNN	Acc = 73–95%
23	Luz 2018^7^	Nr of recordings reported AD n = 214 recordings	n = 184 recordings	AD	SS	Vocalisation, speech rate, number of utterances across discourse event	398	NB	Acc = 68%
24	Martinez de Lizarduy et al 2017^48^	1) AD n = 6, 2) AD n = 20, 3) MCI n = 38	1) n = 12, 2) n = 20 3) n = 62	AD, MCI	SS	Voicing, pauses, F0, harmonicity	18, 40, 100	kNN, SVM, MLP, CNN	Acc = 80–95%
25	Martinez-Sanchez et al 2016^49^	Possible AD n = 45	n = 82	AD	OT	Syllable intervals and their variation	–	–	AUC = 0.87
26	Mirzaei et al 2018^50^	Early AD n = 16, MCI n = 16	n = 16	AD, MCI	OT	HNR, voice length, silences	48	kNN, SVM, DT	–
27	Rentoumi et al 2017^51^	AD n = 30	n = 30	AD	OT	–	–	NB, SVM	Acc = 89%
28	Sadeghian et al 2017^52^	AD n = 26	n = 46	AD	SS	Long pauses, pause and speech duration	65 training, 7 testing	MLP	Acc = 94.4%
29	Satt et al 2013^9^	MCI n = 43, AD n = 27	n = 19	AD, MCI	SS, OT	Verbal reaction time, voiced segments	89	SVM	EER = 15.5–18%
30	Toth et al 2015^53^	MCI n = 32	n = 19	MCI	SS	Pauses, tempo	153 samples from 51 participants	SVM, Random Forest	Acc = 82.4%
31	Toth et al 2018^54^	MCI n = 48	n = 36	MCI	SS	Pausation, tempo and duration	84	NB, Random Forest, SVM	Acc = 75%
32	Warnita et al 2018^55^	AD n = 169	n = 98	AD	SS	Feature set from Interspeech 2010	488 samples from 267 participants	GCNN	Acc = 73.6%
33	Zimmerer et al 2016^56^	AD n = 48	n = 38	AD	SS	Semantic errors, bigram and trigram proportions	–	Logistic regression	–

Abbreviations: Acc, accuracy; AD, Alzheimer’s disease; aMCI, amnestic mild cognitive impairment; AUC, area under curve; CNN, convolutional neural networks; CTree, classification tree; DT, decision tree; EER, equal error rate; GCNN, gated convolutional neural networks; HNR, harmonics-to-noise ratio; kNN, k-nearest neighbor; MCI, mild cognitive impairment; MCI-con, mild cognitive impairment later converted into AD; MCI-non, mild cognitive impairment not converted into AD; MD, mixed dementia; mdMCI, multiple domain mild cognitive impairment; MLP, multilayer perceptron; MLU, mean length of utterance; NB, Naive Bayes; OT, other tasks; POS, part-of-speech; SCI, subjective cognitive impairment; SS, spontaneous speech; SVF, semantic verbal fluency; SVM, support vector machine.

### Study examples

In this section we briefly describe 2 studies to provide the reader with a better understanding of what was examined. These 2 studies are chosen to cover different condition groups, data collection, and analysis methods.

Fraser and colleagues^8^ used the recordings of 264 participants describing the Cookie Theft picture available on DementiaBank corpus. Cookie Theft picture is a commonly used test in language and cognitive disorder assessment because it features a complex scene and describing it triggers diverse language. DementiaBank is a corpus available for research purposes that gathers speech and language data from people with AD and other forms of dementia. The 2 participant groups in Fraser’s study were AD and healthy control group. A total of 370 language and speech features related to part-of-speech, syntactic complexity, grammatical constituents, psycholinguistics, vocabulary richness, information content, and repetitiveness and acoustics categories were extracted. The dataset was divided into test and training data, and machine learning techniques were applied to explore the accuracy of automatic classification between healthy and AD group. Standard accuracy of over 81% was achieved.

Clark and colleagues^35^ included both SVF and PVF tasks from 107 MCI patients and 51 healthy control group participants. The tests were transcribed, and language features, such as the raw count of words, intrusions, repetitions, clusters, switches, mean word frequency, mean number of syllables, algebraic connectivity, and many more were captured. The study paired linguistic measures with the information from magnetic resonance imaging (MRI) scans which allowed creating novel scores. The study concludes that the classifiers trained on novel scores outperformed those trained on raw scores.

### Research questions

The research questions were grouped into 5 categories.

#### What were the characteristics of control and impaired groups?

In 33 studies, 32 different datasets were used. While some studies included up to 3 different datasets for different experiments, a few datasets were used more than once across the studies. The conditions considered in this study were AD and MCI. Although MCI did not feature in the search terms, we decided not to exclude the studies focusing solely on MCI because while MCI patients do not meet the diagnostic criteria of dementia, they can sometimes convert to AD. The studies may therefore provide an insight into the early stages of the disease as well as capture the characteristics of those MCI patients who develop AD and of those who do not. To address the heterogeneity this approach creates, the studies focusing on MCI are looked at separately from the studies concerned with AD detection. Two studies also included other dementia groups (early dementia and mixed dementia) but as both groups only appear once in the dataset, these groups were not included in further analyses.

64% of all studies reported participants’ gender and age. The average number of male participants was 35, and female participants was 50. The number of male and female participants was stated to be balanced in 13 studies and notable differences in the number of male and female participants appeared in 15 studies. There were significant differences in participants’ average age between healthy control (66.94 ± 5.75) and AD group (74.75 ± 4.36), *t*(30) = −4.223, *P* = .000, and between MCI (70.21 ± 5.64) and AD group (74.7*5,* ± 4.36), *t*(25) = −2.351, *P* = .027. The participants’ education level was considered in 45% of the studies. The control group participants had spent, on average, more years in education than the impaired group in all but 1 study where the participants’ education level was considered.

Handedness was controlled for in 2 studies, and all but 4 studies mentioned the language the participants’ spoke.

See Table 2 for participant information.

**Table 2.** Participant information

Participant groups (total number of datasets including the group)	Information variable (number of datasets including this information)	Mean (SD)	Min	Max
**Control group (30)**	*Number of participants (29)*	**42.69 (46.34)**	2	242
	*Age (18)*	**66.94 (5.747)**	57	76
	*Years of education (11)*	**13.44 (2.274)**	9	18
**MCI group (16)**	*Number of participants (15)*	**30.07 (19.41)**	1	83
*Including MCI, aMCI,*	*Age (13)*	**70.21 (5.637)**	57	78
*mdMCI, MCI-con, MCI-non*	*Years of education (7)*	**13.15 (2.353)**	11	16
**AD group (31)**	*Number of participants (27)*	**45.04 (62.68)**	1	242
*Including AD, early*	*Age (14)*	**74.75 (4.360)**	66	80
*AD, intermediate AD, advanced AD, probable AD, possible AD*	*Years of education (8)*	**11.80 (2.006)**	8	15
**Dementia group (2)**	*Number of participants (2)*	**27.00 (15.56)**	16	38
*Including early*	*Age (2)*	**72.74 (8.990)**	66	79
*Dementia, mixed dementia*	*Years of education (1)*	**9.380**	9	9

Abbreviations: AD, Alzheimer’s disease; aMCI, amnestic mild cognitive impairment; MCI, mild cognitive impairment; MCI-con, mild cognitive impairment later converted into AD; MCI-non, mild cognitive impairment not converted into AD; MD, mixed dementia; mdMCI, multiple domain mild cognitive impairment; SD, standard deviation.

#### What kind of language data were collected and how?

From 33 studies, 28 included at least one SS task, 7 studies included a VF task, and 7 studies an OT. 

The aim of SS tasks is to trigger spontaneous speech. This was most often attempted by asking the participants to describe a picture or by engaging in a conversation with the participants. Other tasks used to induce SS included recalling a movie, a day, an event, or a dream. In 1 study, the transcripts from press conferences were used as a source of SS. SS tasks allow analyzing a variety of language attributes, such as word retrieval processes, syntactic, semantic and acoustic impairment, and communication errors.

There are 2 types of VF tasks: PVF and SVF. In the PVF task, the participants are instructed to name as many words as possible in 1 minute that start with the same letter, such as the letter F. In the SVF task, the participants are instructed to name as many words from the same semantic category as possible in 1 minute, such as animals. Traditionally, the measure most commonly used to evaluate performance in fluency tests is the number of total or correct words produced in 1 minute. More recently, NLP has been used for automatic analysis of semantic clusters and SP for the analysis of temporal and acoustic measures.

OT include all the tests that were not concerned with SS or VF, for example, repeating a sentence, reading out a paragraph, writing a story, counting down numbers, pronunciation, or denomination test. These tasks allow for the examination of different aspects of memory, semantic processing, and acoustic and phonetic measures.

In all tests, the language data were audio or video recorded and/or transcribed.

**Figure 2.**
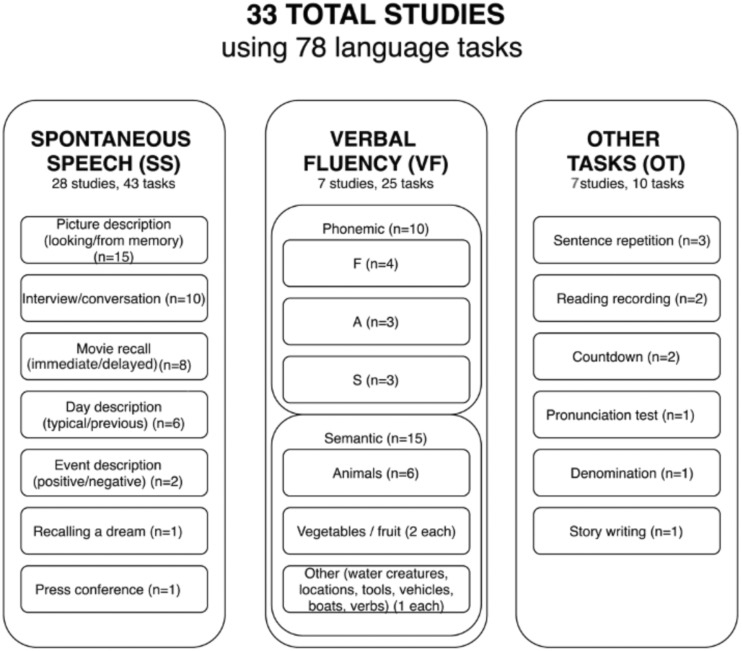
Division of language tests used to identify different health conditions.

Figure 2 provides a summary of the methods and tasks used to collect language and speech data.

#### What language and speech features were the most informative?

The 33 studies included experiments from 21 individual research groups. Out of the individual research groups, 18 included SS tasks and 5 VF and OT tasks. The most informative language and speech features are looked at in 2 categories: those characteristic to AD, and those to MCI.

**Figure 3.**
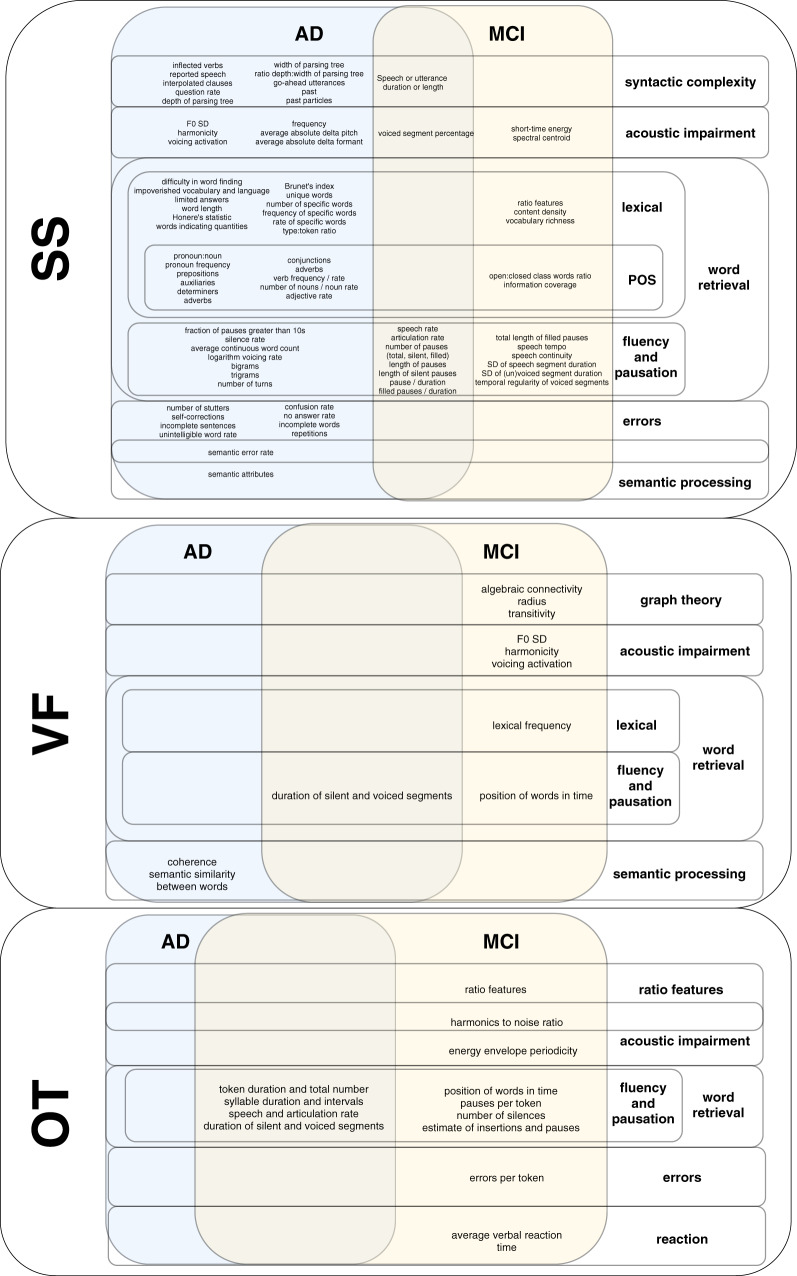
Most informative language and speech features across SS, VF, and OT tasks (AD, Alzheimer’s Disease; MCI, mild cognitive impairment; OT, other tasks; POS, Part-of-Speech; SD, Standard Deviation; SS, spontaneous speech; VF, verbal fluency).

The number of the language and speech features used in the analyses ranged from 4 to 920. As the studies with a large number of features did not report all the features considered, it was difficult to examine what features were studied the most extensively. To avoid the synthesis bias towards the features that have been studied more^57^ and the multiple publication bias of over-representing 1 study or research group with significant results,^58^ each feature that has been reported the most informative by at least 1 research group is reported on equal basis. See Figure 3 for the most informative language features from SS, VF, and OT tasks.

#### What methods were used to classify healthy people and the people with dementia?

Out of 33 studies, 27 used ML to distinguish between healthy people and the people with different medical conditions. Different ML algorithms were used across studies: NNs were used in 17 studies, SVMs in 16, DTs in 11, Naïve Bayes in 7, and logistic regression in 2 studies. See Table 3 for details and definitions.

**Table 3.** Details of ML methods used and the performance achieved. “Average of all reported outcomes” refers to the average of all measures reported across studies using the ML algorithm and performance measure. “Average of best reported outcomes” takes the average measure of the best performance reported in each study (1 measure per study) using the ML algorithm and performance measure

	Classification	AD vs healthy control				CD vs healthy control			
	performance measure	acc (n)	AUC (n)	precision (n)	EER (n)	acc (n)	AUC (n)	precision (n)	EER (n)
Neural Nets (NNs) (n = 17) *NNs are computer systems that are similar in structure to biological neural networks and mimic the way animals learn*^59^	average of all reported outcomes	86% (17)	–	0.64 (4)	–	65% (4)	–	–	–
	average of best reported outcomes	88% (6)	0.96 (1)	0.69 (1)	–	69% (2)	–	–	–
Support Vector Machines (SVMs) (n = 16) *SVMs are supervised models that use training data belonging to 1 or another category, and assign a category to test data based on the training data*^60^	average of all reported outcomes	81% (33)	–	0.68 (4)	14% (2)	78% (13)	–	–	19% (3)
	average of best reported outcomes	88% (9)	–	0.79 (1)	14% (2)	77% (6)	–	–	19% (2)
Decision Trees (DTs) (n = 11) *DTs take several input variables and, based on the observations about them, predict the value of a target variable*^61^	average of all reported outcomes	82% (24)	–	0.63 (5)	–	78% (9)	–	–	–
	average of best reported outcomes	90% (4)	–	0.96 (2)	–	80% (3)	–	–	–
Naïve Bayes (NB) (n = 7) *NB classifiers are simple probabilistic classifiers where each variable contributes independently to the assigning of class label*^62^	average of all reported outcomes	81% (8)	–	0.81 (1)	–	67% (1)	–	–	–
	average of best reported outcomes	81% (4)	–	0.81 (1)	–	67% (1)	–	–	–

Abbreviations: acc, accuracy; AD, Alzheimer’s disease; AUC, area under curve; CD, cognitive decline; EER, equal error rate.

#### What classification performance has been achieved?

The studies reviewed in this paper tend to use different measures to report classification performance (accuracy, precision, Area Under Curve Receiver Operating Characteristics (AUC – ROC), making the comparison of performance difficult. Standard accuracy refers to the level of agreement between the reference value and the test result, and precision refers to the level of agreement between independent test results obtained under stipulated conditions.^63^ ROC curve shows the relationship between clinical sensitivity and specificity for every possible decision threshold. AUC measures the ability of the model to distinguish between the groups for all decision thresholds.

The heterogeneity of the performance measures, as well as the participant groups, data collection, and analysis methods does not allow for a direct comparison of classification accuracy. We aim to tackle this issue in 2 steps. First, we provide a table with qualitative information about the methods that each study concluded to have worked best. Second, as standard accuracy was the most widely used performance measure, we compare the results and the methods used to achieve them in the studies that reported standard accuracy.

Table 4 presents the settings and the approaches that were used when top performance was achieved in each study.

**Table 4.** Most effective technologies

ID	Study	Most effective technologies	Classification performance
1	Ammer and Ayed 2018^30^	feature selection: kNN; classifier: SVM	precision = 79%
2	Beltrami et al 2018^31^	Acoustic features	–
3	Boye et al 2014^32^	–	–
4	Chien et al 2018^33^	bidirectional LSTM RNN	AUC = 0.956
5	Clark et al 2014^34^	Semantic similarity features	–
6	Clark et al 2016^35^	Classifiers with novel scores including MRI data	Acc = 81–84%
7	Fang et al 2017^29^	length of sentence, unique words, non-specific, and specific words	–
8	Fraser et al 2015^8^	Using 35 features	Acc = 82%
9	Garrard et al 2017^28^	Certain scripts and motives	–
10	Gosztolya et al 2016^36^	automatically selected feature set, correlation-based feature selection technique	Acc = 88.1%
11	Gosztolya et al 2019^37^	AD: combination of linguistic and acoustic features; MCI: semantic and acoustic features	Acc = 86%
12	Guinn et al 2014^38^	go-ahead utterances and certain fluency measures	precision = 80%
13	Hernandez-Dominguez et al 2018^39^	AD detection: RFC with coverage and linguistic features; decline detection: RFC with a combination of features with P-value <.001 when correlating with cognitive impairment	Acc = 87–94%
14	Khodabakhsh et al 2014a^40^	SVM, logarithm of voicing ratio, average absolute delta feature of the first formant, and average absolute delta pitch feature	Acc = 88–94%
15	Khodabakhsh et al 2014b^41^	SVM, DT	Acc = 90%
16	Khodabakhsh et al 2015^42^	SVM classifier with the silence ratio feature	Acc = 84%
17	Konig et al 2015^43^	–	EER = 13–21%
18	Konig et al 2018^27^	Fluency tasks	Acc = 86%
19	Lopez-de-Ipina et al 2013a^44^	Including fractal dimension sets	Acc = 75–94.6%
20	Lopez-de-Ipina et al 2013b^45^	SVM and features from 3 datasets: spontaneous speech, emotional response and energy features	Acc = 93.79%
21	Lopez-de-Ipina et al 2015^46^	MLP for Katz’s and Castiglioni’s algorithm with a window-size of 320 points	Acc = 95%
22	Lopez-de-Ipina et al 2018^47^	SS task and AD patients: the recording environment within a relaxing atmosphere; the presence of subtle cognitive changes in the signal due to a more open language; and the use of AD patients instead of MCI subjects.	Acc = 73–95%
23	Luz 2018^7^	–	Acc = 68%
24	Martinez de Lizarduy et al 2017^48^	spontaneous speech task; CNN	Acc = 80–95%
25	Martinez-Sanchez et al 2016^49^	The standard deviation of the duration of ΔS	AUC = 87%
26	Mirzaei et al 2018^50^	kNN with 18 features	–
27	Rentoumi et al 2017^51^	–	Acc = 89%
28	Sadeghian et al 2017^52^	using all the potential features, including and choosing the 5 most informative ones: 1) MMSE, 2) race, 3) fraction of pauses greater than 10s, 4) fraction of speech length that was pause, 5) words indicating quantities	Acc = 94.4%
29	Satt et al 2013^9^	Using 20 features	EER = 15.5–18%
30	Toth et al 2015^53^	SVM with manually extracted features	Acc = 82.4%
31	Toth et al 2018^54^	RFC with automatic and significant feature set	Acc = 66.7–75%
32	Warnita et al 2018^55^	10-layer CNN with Interspeech 2010 feature set	Acc = 73.6%
33	Zimmerer et al 2016^56^	connectivity, closed-class words, semantic error rate	–

Abbreviations: Acc, accuracy; AD, Alzheimer’s disease; CNN, convolutional neural networks; DT, decision trees; ET, emotional temperature; kNN, k-nearest neighbor; LSTM RNN, long short-term memory recurrent neural network; MLP, multilayer perception; MMSE, mini-mental state examination; MRI, magnetic resonance imaging; RFC, random forest classifier; SVM, support vector machine.

Standard accuracy was used as a classification performance measure in 17 tasks across 15 studies that aimed to distinguish the people with AD from the people without AD and in 8 studies looking at MCI. The average classification accuracy was significantly lower when detecting MCI (81.7% ± 5.3%) than when detecting AD (88.9% ± 8.0%), *t* (14) = 2.40, *P* = .031.

Top result in AD detection (95% classification accuracy) was achieved using an SS task to collect information about voiced and unvoiced segments and other acoustic and phonetic features. Lopez-de-Ipina et al used NN to distinguish the people with AD from those without AD.^46–47^

Top result in MCI detection (86% classification accuracy) was reached by Konig et al^27^ using SVF and PVF to collect language data, SP to analyze the data, and SVM to discriminate between the people with and without MCI.

## DISCUSSION

We found that the sociodemographic variables often differ between healthy and impaired groups, especially age. The language data were usually collected using SS tasks, with the most informative language features falling under lexical, syntactic, semantic, and acoustic impairment. NNs, SVMs, and DTs performed well as classifiers; 89% average classification accuracy was reached in AD detection and 82% in MCI detection.

### Synthesis

The majority of the studies reviewed in this article demonstrate promising results in identifying AD or MCI based on speech and language data. While the results are promising, there is also room for improvement. For example, age, gender, education level, and handedness can affect speech and the outcome of language tests. However, there were significant differences in participants’ ages between healthy and AD groups, more female than male participants were included in the studies, people with a clinical condition tended to be less educated than the control group, and only 6% of the studies considered whether the participants were right- or left-handed. Similarly, the majority of participants spoke European languages, leading to very few non-European languages being considered.

Two popular and well-performing language tasks were SS and VF. Promising results were achieved using language features relating to word retrieval, semantic and acoustic impairment, and error rate.

Various ML algorithms were used to classify between different condition groups. The best performing models were NNs, SVMs, and DTs.

The measures used to report performance were heterogeneous, making the comparison of the technologies difficult. Focusing on the studies that used accuracy as a metric, we found that the highest classification accuracy was achieved using SS task, SP method, and NN classifiers when distinguishing between AD and healthy groups, and VF task, SP method, and SVM classifier when detecting MCI. Average classification accuracy was 89% in AD and healthy group distinction, and 82% in MCI detection.

### Recommendations for future research

Based on the findings of this study, we propose the following:

1. We encourage future research to construct demographically and socioeconomically balanced datasets to minimize the effect of age and other factors on the results.
2. We suggest including a larger number of participants to allow more data to be used when training a machine learning model.
3. We recommend including non-European languages in future studies as the vast majority of the studies so far have been conducted in European languages.
4. Early detection of dementia could benefit from longitudinal studies concerned with MCI to examine the language of those participants who convert from MCI to AD and of those who do not. This approach was taken in Clark and colleagues.^35^
5. In future studies, we suggest integrating linguistic analysis and signal processing to achieve maximum accuracy. Most studies focus on either SP and acoustic features or NLP and linguistic features. However, most language tasks are audio recorded which would allow collecting both acoustic and linguistic data (using both audio samples and transcripts). We suggest that adding linguistic variables (lexical, semantic, syntactic) to SP approach, and vice versa, adding SP measures (acoustic, voiced and unvoiced segment analysis) to studies mainly focusing on linguistic features. This will allow for the expansion of the set of variables beneficial in ML approach and could lead to more accurate classification results. An example of a study that has used both acoustic and linguistic measures was conducted by Fraser and colleagues.^8^
6. The reviewed papers use slightly different metrics to measure the performance making it difficult to compare. We recommend using the 4 standard measures: Accuracy, Precision, Recall, F1-score. AUC can be used in addition to those 4.

The studies reviewed in this article also include 19 suggestions for future research: 1) ensure standardized recordings and language samples, 2) add new and challenging tasks, 3) calibrate audio measurements, 4) add new features, 5) couple speech analysis with neuroimaging, 6) include follow-up studies, 7) conduct longitudinal studies, 8) add linguistic and acoustic features, 9) automate feature selection, 10) include voice onset time, 11) extend the number of MCI samples, 12) research the effect of sample size in healthy control groups, 13) perform cross-linguistic studies, 14) use automatic transcription of language tasks, 15) include nonverbal communication (gestures), 16) include syllable-timed and low-resource languages, 17) replicate the results of currently available studies, 18) evaluate the temporal change and the severity of the disease, and 19) include more forms of dementia, such as vascular dementia.

### Study limitations

To evaluate the limitations and establish the confidence level of the outcomes, we adapt GRADE guidelines.^64^

There are 5 main limitations, 4 of which contributed to the decision to rate down the outcome confidence level from high to moderate.

First, the chance of publication bias must be acknowledged, meaning that only the studies with more significant results might have been published.^65^^,^^66^ Although publication bias was undetected in the current review, it is especially common in literature reviews written in the early stages of the specific research area due to negative studies being delayed^66^ and should therefore be mentioned. Potential publication bias was not used to decrease the confidence level.

Second, there is a potential synthesis bias in the study location, as only articles written in English were included.^57^^,^^58^ This did not allow for the data available in other languages to be considered, limiting our dataset and possibly contributing to the small number of non-European languages being included. Language bias can especially affect the outcomes relating to the most informative language features, as these are directly dependent on the language used.

Third, there is a risk of bias in the outcomes of studies focusing on AD detection because the AD group was very often significantly older than the control group. This increases the chance of the most informative language features being characteristic to older age instead of AD, as well as the classification algorithms differentiating between older and younger, and not necessarily detecting AD.

Fourth, there is a risk of bias when reporting the outcomes of the studies concerned with MCI. The fact that our search terms did not include MCI is likely to have led to a situation where additional studies did exist—but were inaccessible to us—and therefore did not get included in the analysis.

Fifth, there is a potential risk of bias in reporting the classification performance, as often only the best outcomes are included, potentially leading to skewed understanding of how well the algorithms worked.

The last 4 limitations contributed to the confidence levels of our outcomes concerned with informative language features, classification algorithms, and classification performance to decrease from high to moderate.

## CONCLUSION

In this systematic review on automatic AD detection from speech and language, we report the characteristics of healthy and impaired groups, summarize the language tests that have been used, present the language and speech features that have shown to be the most informative, and identify the machine learning algorithms used and the classification performance achieved.

Our findings show that the balance in the demographic variables across dementia and healthy groups could be improved. We also found that studies looking at SS have achieved top accuracy in distinguishing between AD and healthy conditions. Informative language and speech features capture problems with word retrieval, semantic processing, acoustic impairment, and errors in speech and communication. From ML algorithms, NNs and SVMs were the most widely used, and top accuracy was also achieved with these models. Standard accuracy was the most common metric used to report the classification performance, with the average accuracy in AD detection being 89%, and in MCI detection 82%.

In the future studies, we suggest standardizing the metrics used to report classification performance, focusing on MCI and the early stages of dementia to contribute to early detection, combining signal processing and linguistic information, including non-European languages, and constructing larger and more demographically balanced datasets.

## FUNDING

This work was supported by the Economic and Social Research Council Cambridge Doctoral Training Partnership (DTP) grant number ES/P000738/1.

## AUTHOR CONTRIBUTIONS

SB and AK contributed to the conception of the manuscript. UP performed article collection and examination, data summarization and analysis, and drafted the manuscript. SB contributed significantly to article screening and data analysis and revised and edited the manuscript. AK provided research direction, commented on the manuscript, and approved the final version of the manuscript.

## CONFLICT OF INTEREST STATEMENT

None declared.
